# Evaluation of drug-induced liver toxicity of trovafloxacin and levofloxacin in a human microphysiological liver model

**DOI:** 10.1038/s41598-023-40004-z

**Published:** 2023-08-16

**Authors:** Tim Kaden, Katja Graf, Knut Rennert, Ruoya Li, Alexander S. Mosig, Martin Raasch

**Affiliations:** 1 Dynamic42 GmbH, Jena, Germany; 2 Institute of Biochemistry II, Center for Sepsis Control and Care, Jena University Hospital, Jena, Germany; 3 Biopredic International, St Gregoire, France

**Keywords:** Cell biology, Biotechnology, Tissue engineering, Drug discovery, Drug safety, Drug screening, Toxicology

## Abstract

Drug-induced liver injury induced by already approved substances is a major threat to human patients, potentially resulting in drug withdrawal and substantial loss of financial resources in the pharmaceutical industry. Trovafloxacin, a broad-spectrum fluoroquinolone, was found to have unexpected side effects of severe hepatotoxicity, which was not detected by preclinical testing. To address the limitations of current drug testing strategies mainly involving 2D cell cultures and animal testing, a three-dimensional microphysiological model of the human liver containing expandable human liver sinusoidal endothelial cells, monocyte-derived macrophages and differentiated HepaRG cells was utilized to investigate the toxicity of trovafloxacin and compared it to the structurally-related non-toxic drug levofloxacin. In the model, trovafloxacin elicited vascular and hepatocellular toxicity associated with pro-inflammatory cytokine release already at clinically relevant concentrations, whereas levofloxacin did not provoke tissue injury. Similar to in vivo, cytokine secretion was dependent on a multicellular immune response, highlighting the potential of the complex microphysiological liver model for reliably detecting drug-related cytotoxicity in preclinical testing. Moreover, hepatic glutathione depletion and mitochondrial ROS formation were elucidated as intrinsic toxicity mechanisms contributing to trovafloxacin toxicity.

## Introduction

Drug-induced liver injury (DILI) is one of the most common adverse events terminally leading to drug withdrawal from commercial markets^1,2^. In this context, 76 commercially available drugs were withdrawn in the United States between 1969 and 2002, of which 12 were associated with hepatic adverse events^3^. Epidemiological studies conducted in Iceland and France suggested an annual incidence rate of DILI between 13.9 and 19.1 per 100,000 patients^4,5^. More recent studies, however, have shown an even larger variation between 2.7 and 32.8 cases per 100,000 patients^6–8^. These differences can be attributed to different populations, divergent study methodologies and lack of uniform diagnostic criteria, which complicate an exact concretization of the incidence rate. DILI constitutes the primary cause of acute liver failure in the United States and is associated with the occurrence of severe clinical manifestation, frequently requiring subsequent liver transplantation and low patient survival rates^9,10^.

Approximately 50% of the acute liver failure cases were related to the administration of the analgesic drug acetaminophen in the United States^11^ and between 40 and 70% in Europe and Great Britain^12^. Acetaminophen-induced hepatotoxicity is classified as intrinsic DILI and is concentration-dependent and predictable^13^. In contrast, idiosyncratic DILI is discriminated from intrinsic DILI by a lesser degree of drug dose dependency and underlies a high variability of patient susceptibility and multifactorial risk factors, such as age, gender, sex, genetic predisposition and environmental influences^14,15^.

Prospective studies in the United States identified antimicrobial medication as one of the major drug classes involved in the development of idiosyncratic DILI^16,17^. This drug class includes the third-generation fluoroquinolone antibiotics trovafloxacin (TVX) and levofloxacin (LVX). TVX was withdrawn from the market in 1999 after the occurrence of 140 severe adverse hepatic events and 14 cases of acute liver failure in human patients^18^, without indicating these side effects in mice studies^19^. The structurally-related compound LVX was classified as a generally well tolerated non-DILI drug^20^ associated with a substantially lower reporting rate of liver adverse effects compared to TVX^21^.

To uncover the occurrence of DILI even before human clinical trials, rodents and non-rodents are considered as indispensable preclinical model organisms to test drug safety and hepatic adverse events. Notwithstanding, recent studies have shown that current animal models have several limitations in predicting liver toxicity in humans^22,23^. Therefore, great effort has been put into the development of relevant human-based in vitro models of the liver. Within this scope, TVX-induced hepatotoxicity was previously detected and discriminated from its non-toxic analogue LVX in in vitro models, for example in bioprinted 3D primary liver tissue^24^, 3D spheroid co-culture of primary human hepatocytes and Kupffer cells^25^ and human liver microphysiological platforms^26,27^. However, non-physiological microarchitecture, inclusion of non-native cells, absence of immune cells, modified drug application or supraphysiological drug concentrations were limitations within these studies.

We previously described the establishment and characterization of a microphysiological liver model containing human umbilical cord vein endothelial cells (HUVECs), monocyte-derived macrophages (MDMs) and HepaRG hepatocytes^28^. Herein, defined expression of the hepatocyte polarization markers zonula occludens-1 (ZO-1), asialoglycoprotein receptor 1 (ASPGR1) and multidrug resistance-associated protein-2 (MRP-2) as well as stable urea and albumin synthesis were shown. Further analysis demonstrated morphological similarities of cultured HepaRG compared to human primary hepatocytes.

To further increase the physiological relevance and to adapt the model for drug testing, this study used expandable upcyte liver sinusoidal endothelial cells (LSECs) instead of HUVECs, which retain morphology and functionality observed in primary human LSECs^29^. Leveraging the microphysiological liver model, we investigated the toxicity of TVX at concentrations relatable to daily therapeutic doses in human patients. To mimic the transportation route of an orally administered drug via the sinusoidal blood flow, the drug compounds were perfused through the vascular cultivation chamber. Treatment of the liver model with TVX resulted in decreased cellular viability with concomitant decrease in cell-specific fluorescence markers. Furthermore, increased release of cell damage markers lactate dehydrogenase (LDH) and alanine aminotransferase were detected. Elevated levels of pro-inflammatory cytokines were measured in TVX-treated liver models indicating an inflammatory response. Augmented glutathione depletion and accumulation of mitochondrial reactive oxygen species (ROS) in hepatocytes were suggested as intrinsic toxicity mechanisms of TVX. In contrast, the administration of the related non-toxic drug LVX did not elicit commensurable DILI.

## Results

### Liver model conceptualization and application for drug toxicity testing

**Figure 1:**
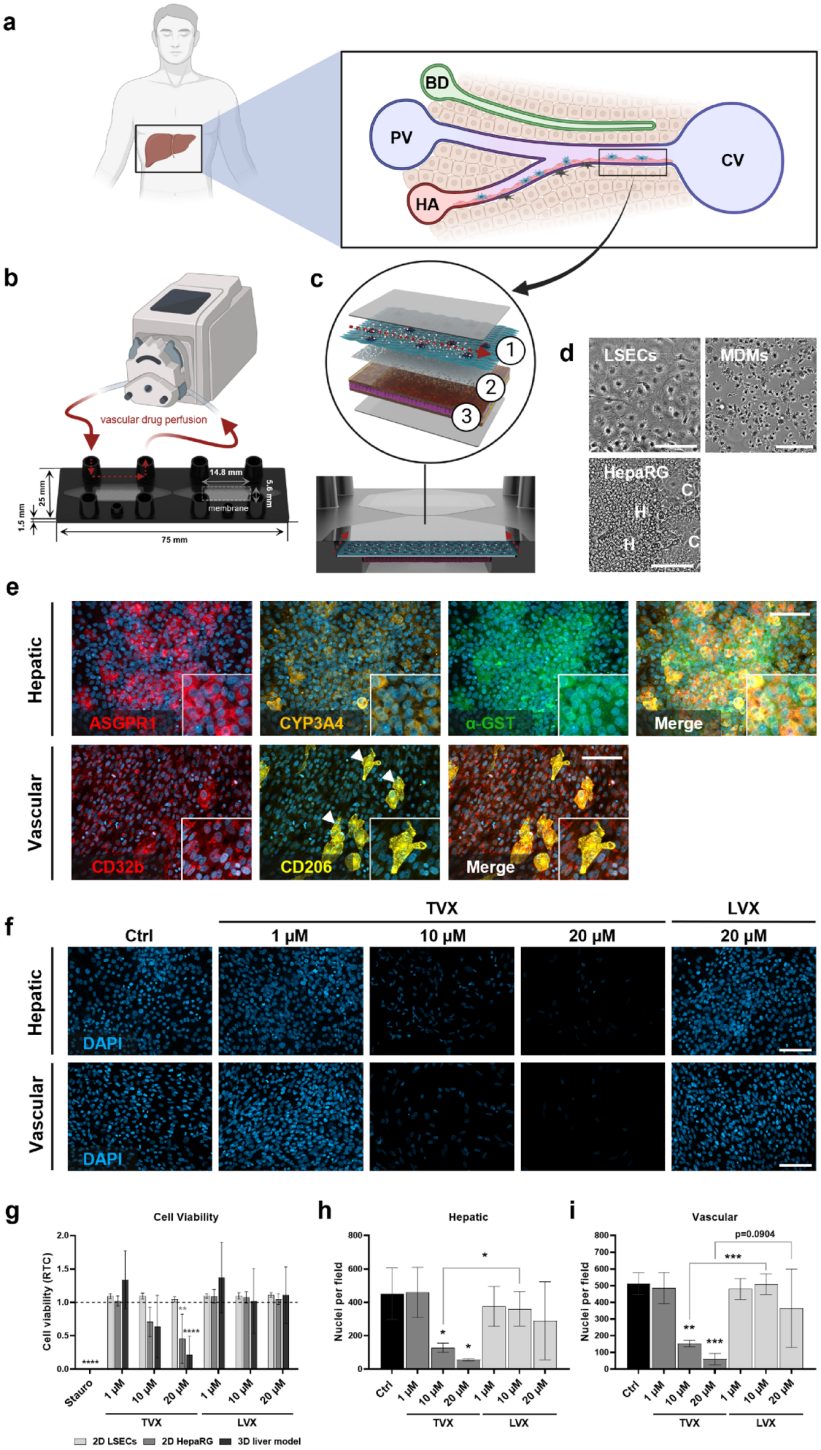
Assembly, morphological analysis and drug treatment of the liver model. (**a**) Localization of the human liver and enlarged schematic representation of the human liver sinusoid comprising the afferent portal venule (PV), the hepatic arteriole (HA) and the efferent bile duct (BD). Both portal venule and hepatic arteriole channel the sinusoidal bloodstream towards the efferent central vein (CV). The liver sinusoid is composed of hepatocytes (brown) and non-parenchymal cells, such as LSECs (red), KCs (blue) and hepatic stellate cells (black). Parenchymal and non-parenchymal cell types are separated by the space of Disse. (**b**) Technical setup of Dynamic42 biochips under unidirectional vascular perfusion (red arrows) applied by an external peristaltic pump. Biochip dimensions and membrane specifications are illustrated in millimeter (mm) (**c**) Emulation of a liver sinusoidal cross-section in the Dynamic42 biochip. Enlarged cross-sectional view of the Dynamic42 biochip platform with both culture chambers, porous membrane, and incorporated cells. Upcyte LSECs (light blue) and human MDMs (dark blue) resemble a confluent vascular layer in the upper chamber (1). Differentiated HepaRG cells (pink) form a hepatic layer in the opposing lower chamber (3). A porous membrane (grey) recapitulates the space of Disse and separates both chambers (2). The direction of the vascular perfusion is indicated in the figure by red arrows. Figures were created with BioRender.com. (**d**) Brightfield images of LSECs, MDMs and differentiated HepaRG cells, consisting of hepatocytes (H) and cholangiocytes (C), prior to seeding into biochips. Scale bar, 100 µm. (**e**) Immunofluorescence staining of control-treated hepatic and vascular cell layers in the liver model after 7 days of perfusion. Representative images of hepatic layers stained for ASGPR1 (red), CYP3A4 (orange), α-GST (green) and vascular layers (LSECs/ MDMs) stained for CD32b (red) and CD206 (yellow). Counterstaining of nuclei with DAPI (blue). White arrow heads indicate MDMs in the vascular layer. Scale bar, 100 µm. (**f**) Representative fluorescence images of liver models after 7 days of treatment with: control (Ctrl, 0.1% DMSO), TVX at 1 µM, 10 µM, 20 µM and LVX at 20 µM for 7 days. Cell nuclei from hepatic and vascular layers were stained for DAPI (blue). Scale bars, 100 µm. (**g**) Measurement of cellular viability in 2D cultured HepaRG cells, LSECs, and 3D liver models. Viability assay performed with 2D cultures or liver models treated with control (Ctrl, 0.1% DMSO), 10 µM staurosporine (Stauro), TVX and LVX at a concentration of 1 µM, 10 µM or 20 µM for 7 days. Cell viability was measured as relative light units (RLU). Bars represent mean plotted as ratio to control (RTC, dotted baseline) ± SD of at least 3 independent experiments (n ≥ 3). (**h**, **i**) Quantification of nuclei counts per image in hepatic (**h**) and vascular (**i**) cell layers after 7 days of treatment. Bars represent mean ± SD of 3 independent experiments (n = 3 chips with 5 randomly selected membrane regions for each condition). *p ≤ 0.05, **p ≤ 0.01, ***p ≤ 0.001 (Two-way ANOVA with Tukey’s multiple comparison test (1g) or One-way ANOVA with Dunnett’s multiple comparison test, two-tailed t test for comparison between indicated conditions(1h-i)).

Liver models were conceptually adapted to the human liver sinusoidal architecture (Fig. 1a) and were sequentially assembled in a perfused biochip platform (Fig. 1b) by an endothelial layer of LSECs, followed by MDMs and differentiated HepaRG cells (Fig. 1c and d). Cellular growth and morphology were evaluated continuously by light microscopy throughout the study (Fig. S1). The expression of hepatic markers ASGPR1, cytochrome P450 3A4 (CYP3A4) and alpha-glutathione s-transferase (α-GST) were demonstrated by immunofluorescence staining (Fig. 1e upper panel). Expression of ASGPR1, a specific hepatocyte differentiation marker located dominantly at basolateral and sinusoidal membranes, but not at the bile canalicular membrane^28,30^, was observed in hepatocytes indicating appropriate cellular differentiation of HepaRG cells after 7 days of perfusion. Apart from the expression on cell membranes, cytoplasmic localization of ASGPR1 in hepatocyte-like cells was further shown, which has already been described for human liver tissue^31^. In the same study, more diffuse ASGPR1 expression patterns were shown for human hepatocellular carcinoma. ASGPR1 negative cells were considered as cholangiocytes. Moreover, the majority of hepatocytes preserved the expression of the drug metabolizing enzyme CYP3A4 over 7 days, as previously shown^32^. In addition, the expression of cytoplasmic α-GST refers to the antioxidative capacity of hepatocytes to detoxify reactive electrophiles^33^. Furthermore, integrity of LSECs was confirmed by perinuclear expression of Fc fragment of IgG receptor IIb (CD32b) (Fig. 1e lower panel). CD32b is an uptake receptor, which is mainly expressed by LSECs and minorly by Kupffer cells (KCs)^29,34^. The presence of MDMs was proven by expression of mannose receptor (CD206), which is also expressed by LSECs to a lesser extend^29,35^ (Fig. 1e lower panel). The use of controls without primary antibodies further demonstrated that the staining of vascular and hepatic cell markers was indeed specific (Fig. S2).

Liver models were treated with the indicated concentrations of TVX, LVX and staurosporine for 7 days and were monitored by light microscopy for cellular integrity and confluency on day 0, 3 and 7 (Fig. S3). Disruption of layer integrity in staurosporine-treated and TVX-treated models was already detected on day 3, which was more clearly manifested by cell elongation (white arrow heads) and detachment (white asterisks) on day 7. In contrast, cell layers of control-treated and LVX-treated models maintained their cell layer confluency over the treatment period of 7 days.

In addition, TVX treatment resulted in a concentration-dependent reduction of DAPI-positive nuclei in both hepatic and vascular chambers compared with control treatment and treatment with LVX (Fig. 1f). This clearly demonstrates the cytotoxic effect of TVX, which leads to detachment of nuclei from the membrane after a treatment period of 7 days. At low concentrations of 1 µM TVX, the cytotoxic effect was not observed. Further measurement of cellular viability (Fig. 1g) and quantification of nuclei numbers per field (Fig. 1h and i), revealed a concentration-dependent decrease in viability and nuclei after treatment with TVX. Precisely, cellular viability was significantly decreased by 78% of control-treated models after treatment with 20 µM TVX, whereas the positive control staurosporine completely reduced the cellular viability to zero (Fig. 1g). Daily treatment with 10 µM TVX resulted in a 49% decrease in cellular viability. No significant alterations of the cellular viability were observed for LVX treatment compared to control models.

Treatment of 2D cultured HepaRG cells with TVX similarly resulted in a concentration-dependent reduction in cell viability (Fig. 1g), suggesting a direct and intrinsic hepatotoxicity of the drug. Administration of 20 µm TVX induced a significant reduction in cellular viability in 2D cultured HepaRG after 7 days. Comparison of TVX-induced toxicity in 2D cultured HepaRG and 3D liver models demonstrated a slightly higher decrease in viability in 3D models when treated with 20 µm TVX. No significant differences between 2D HepaRG cultures and 3D models were observed at lower TVX concentrations. Interestingly, administration of TVX to 2D cultured LSECs did not result in decreased viability at all TVX concentrations, indicating that the toxicity in the 3D model is primarily due to direct injury of HepaRG cells and subsequently, as a secondary response, damage to LSECs. Application of LVX to either 2D or 3D models did not elicit changes in cell viability compared to control-treated models.

In 3D models, treatment with 10 µM and 20 µM TVX further resulted in a significant reduction of nuclei counts in both hepatic (Fig. 1h) and vascular layers (Fig. 1i) in relation to control-treated models. Cytotoxicity of TVX was not limited to the hepatic side and was also observed for non-parenchymal cell types of the vascular layer. The administration of 10 µM TVX in the model resulted in a significant decline in nuclei counts in hepatic and vascular cell layers compared to its analog LVX. A minor reduction in nuclei counts was also observed in hepatic and vascular layer of models treated with 20 µM LVX, but was considerably lesser in contrast to TVX-treated models (Fig. 1h and i).

### Examination of vascular and hepatic fluorescence marker expression and morphology in drug-treated liver models

To assess the effect of TVX and LVX on vascular and hepatic tissue morphology, immunofluorescence staining and signal quantification was performed 7 days after drug treatment. Liver models were treated by repeated daily dosing with vehicle control and TVX or LVX at 1 µM, 10 µM and 20 µM.

**Figure 2:**
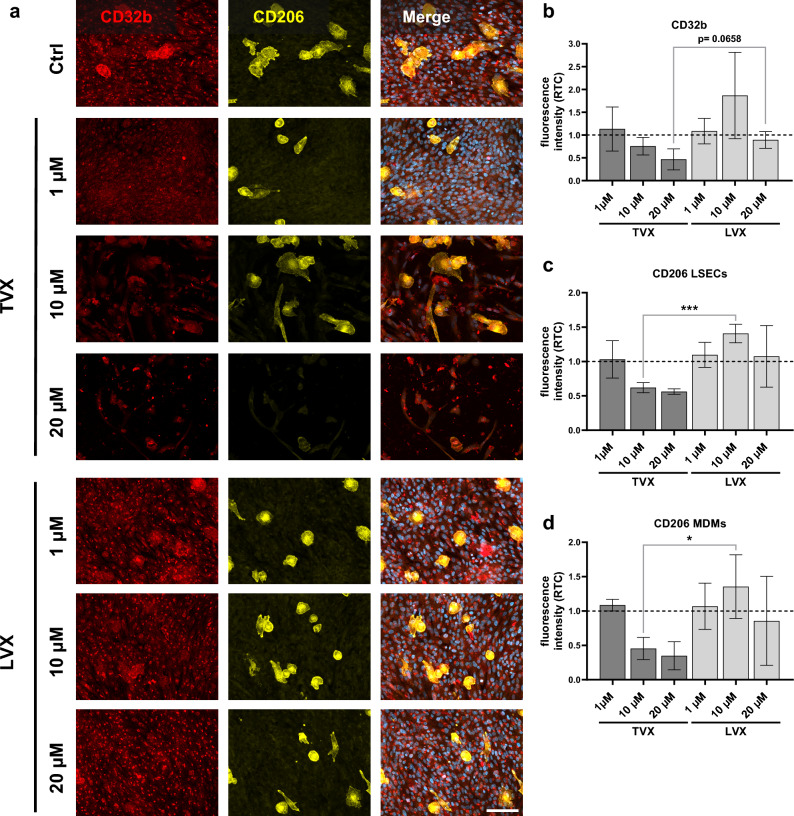
Morphological analysis of vascular cell layers in the liver model. (**a**) Representative immunofluorescence images of vascular layers (LSECs/ MDMs) stained for CD32b (red), CD206 (yellow) and nuclei (DAPI, blue in merge image) after treatment with control (Ctrl, 0.1% DMSO), TVX and LVX at a concentration of 1 µM, 10 µM or 20 µM for 7 days. Scale bar, 100 µm. (**b**–**d**) Signal quantification of CD32b (**b**), CD206 LSECs (**c**) and CD206 MDMs (**d**) fluorescence intensities. Bars show fluorescence intensity plotted as ratio to control (RTC, dotted baseline) and represent mean ± SD of 3 independent experiments (n = 3 with 5 randomly selected membrane regions for each condition). *p ≤ 0.05, ***p ≤ 0.001 (One-way ANOVA with Dunnett’s multiple comparison test, two-tailed t test for comparison between indicated conditions).

Stimulation of the liver model with TVX resulted in increased cellular injury in the vascular layer, as shown by a reduction of cell nuclei in merged representative fluorescence images (Fig. 2a). In contrast, no morphological alterations of LSECs or MDMs compared to control-treated models were observed after LVX treatment. Treatment with TVX induced a decreasing trend of the vascular markers CD32b and CD206 (Fig. 2b, c and d) in relation to control-treated models. Compared with LVX, 10 µM TVX reduced the expression of CD206 in LSECs and MDMs significantly (Fig. 2c and d).

**Figure 3:**
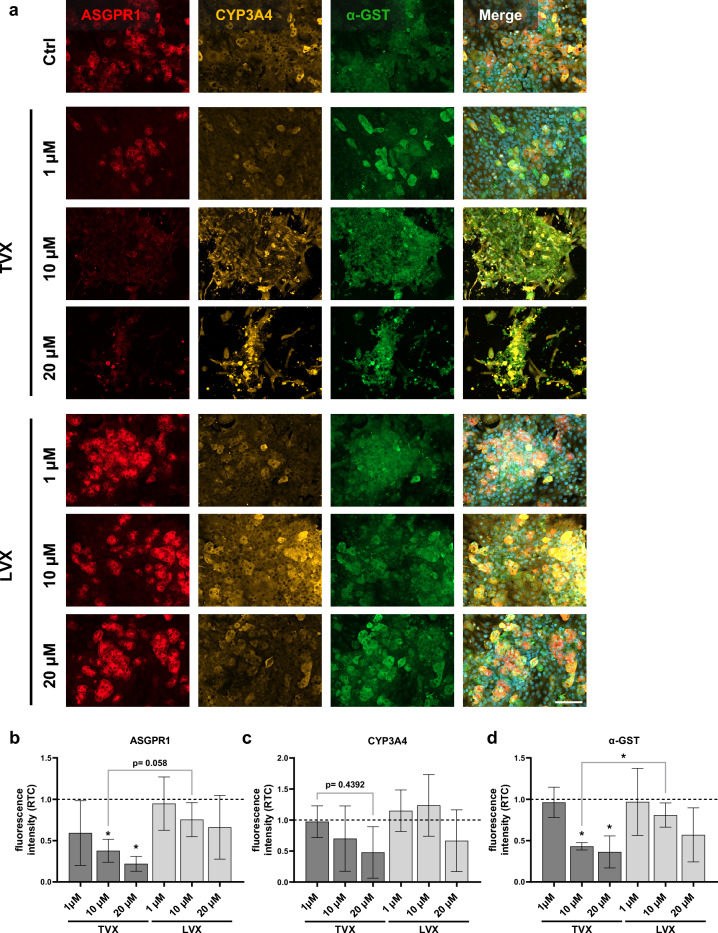
Morphological analysis of hepatic layers in the liver model. (**a**) Representative immunofluorescence images of hepatic layers (HepaRG) stained for ASGPR1 (red), CYP3A4 (orange), α-GST (green) and nuclei (DAPI, blue in merge image) after treatment with control (Ctrl, 0.1% DMSO), TVX and LVX at a concentration of 1 µM, 10 µM or 20 µM for 7 days. Scale bar, 100 µm. (**b-d**) Signal quantification of ASGPR1 (**b**), CYP3A4 (**c**) and α-GST (**d**) fluorescence intensities. Bars show fluorescence intensity plotted as ratio to control (RTC, dotted baseline) and represent mean ± SD of 3 independent experiments (n = 3 with 5 randomly selected membrane regions for each condition). *p ≤ 0.05 (One-way ANOVA with Dunnett’s multiple comparison test, two-tailed t test for comparison between indicated conditions).

Morphological analysis of hepatocellular layers indicated the occurrence of marked toxicity after treatment with TVX concentrations ≥ 10 µM for 7 days in accordance with vascular injury. This was manifested in a loss of tissue integrity and delocalization of ASGPR1, CYP3A4 and α-GST fluorescence signals (Fig. 3a). Further, induction of toxicity by TVX resulted in loss of the organotypic trabecular arrangement of differentiated hepatocytes and a subsequent change towards an elongated morphology. A drop of hepatocyte cell counts was indicated by decreasing DAPI-positive nuclei in merged images. In addition to the observed morphological changes, TVX treatment resulted in a concentration-dependent reduction of hepatic ASGPR1 (Fig. 3b) and α-GST (Fig. 3d) expression. In comparison to LVX, treatment with 10 µM TVX significantly decreased ASGPR1 and α-GST signal intensities. CYP3A4 signal intensities demonstrated a downward tendency after exposition of the liver model to 10 µM and 20 µM TVX (Fig. 3c). LVX treatment further resulted in a concentration-dependent decline in the fluorescence intensity of ASGPR1, CYP3A4 and α-GST, especially at 20 µM (Fig. 3b, c and d). However, the typical hepatocyte morphology and tissue integrity illustrated in the representative images was not affected.

### Analysis of effluent toxicity markers LDH and ALT in drug-treated liver models

**Figure 4:**
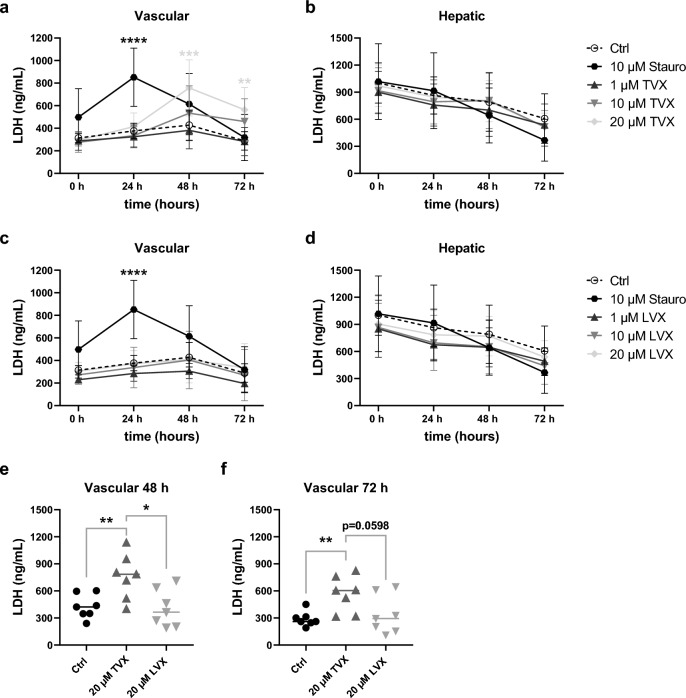
Detection of released LDH in the liver model. (**a**–**d**) LDH release in vascular and hepatic supernatants of TVX-treated (**a**, **b**) and LVX-treated (**c**, **d**) models at various conditions: control (Ctrl, 0.1% DMSO), 10 µM staurosporine (Stauro), TVX and LVX at a concentration of 1 µM, 10 µM or 20 µM for up to 72 h in the liver model. (**e**, **f**) Comparison of LDH release in vascular supernatants 48 h (**e**) and 72 h (**f**) after treatment with TVX or LVX (20 µM). The quantified LDH concentration (ng/mL) was plotted as mean ± SD of at least 5 independent experiments (n ≥ 5). *p ≤ 0.05, **p ≤ 0.01, ***p ≤ 0.001, ****p ≤ 0.0001 (Two-way ANOVA with Dunnett’s multiple comparison test (**a**–**d**), two-tailed t test (**e**–**f**)).

**Figure 5:**
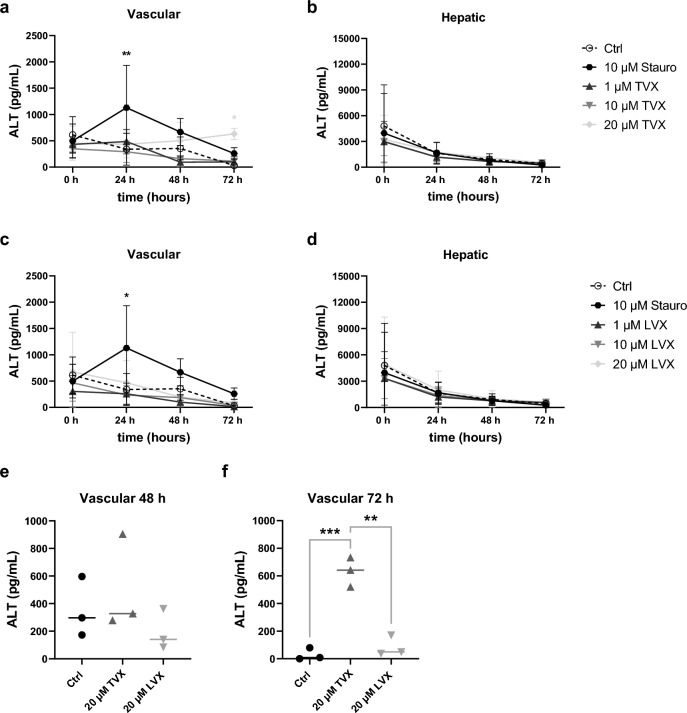
Determination of ALT in the liver model. (**a-d**) ALT release in vascular and hepatic supernatants of TVX-treated (**a-b**) and LVX-treated (**c-d**) models at various conditions: control (Ctrl, 0.1% DMSO), 10 µM staurosporine (Stauro), TVX and LVX at a concentration of 1 µM, 10 µM or 20 µM for up to 72 h in the liver model. (**e–f**) Comparison of ALT release in vascular supernatants 48 h (**e**) and 72 h (**f**) after treatment with TVX or LVX (20 µM). The quantified ALT concentration (pg/mL) was plotted as mean ± SD of 3 independent experiments (n = 3). *p ≤ 0.05, **p ≤ 0.01, ***p ≤ 0.001 (Two-way ANOVA with Dunnett’s multiple comparison test (**a-d**), Two-tailed t test (**e–f**)).

To validate DILI by relevant toxicity markers, medium supernatants from vascular and hepatic biochip chambers were analyzed for the cell damage marker LDH (Fig. 4) and hepatic injury marker ALT (Fig. 5). Treatment with 20 µM TVX resulted in a concentration-dependent increase in LDH levels after stimulation at the vascular cell layer for 48 h and 72 h (Fig. 4a), but not at the hepatic compartment of the model (Fig. 4b). The peak of LDH release in models treated with 20 µM TVX was observed after 48 h, while the positive control staurosporine already reached a significantly increased LDH release after 24 h. A closer comparison revealed a significant rise of LDH in models treated with 20 µM TVX compared to the control and models stimulated with 20 µM LVX after 48 h (Fig. 4e) and 72 h (Fig. 4f). An increasing trend in LDH was further observed for 10 µM TVX compared to control-treated models after 48 h and 72 h (Fig. 4a). No elevations in LDH release at the vascular or hepatic side were observed in response to LVX treatment (Fig. 4c and d). Apart from that, ALT activity was measured in vascular and hepatic cell culture supernatants of liver models treated for up to 72 h. The highest concentration of TVX elicited a significant increase in hepatocyte-specific ALT release after 72 h compared to control-treated models, whereas the positive control staurosporine induced a significant rise of ALT after 24 h (Fig. 5a). Interestingly, increased ALT release was mainly detected in the vasculature of the model. In addition, the increase in ALT upon TVX administration was significantly elevated compared to LVX at 20 µM (Fig. 5f). Treatment with LVX did not affect ALT release in the vascular chamber compared to the control (Fig. 5c). No considerable effects were observed for either TVX or LVX in the hepatic chamber (Fig. 5b and d), which was consistent with the results of the LDH measurement. The observed accumulation of LDH and ALT in vascular medium supernatants compared to supernatants from the static hepatic chamber could be attributed to increased permeability upon cytotoxicity and subsequent accumulation of toxicity markers in the vascular perfusion (Supplementary Fig. S4). Moreover, low permeability was further shown in confluent co-culture models of LSECs and HepaRG hepatocytes.

### Investigation of drug-induced immunomodulatory effects in liver models by measurement of vascular cytokine levels

**Figure 6:**
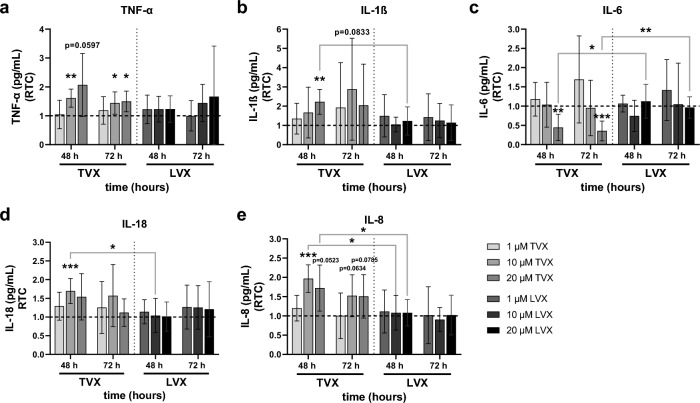
Cytokine release in vascular supernatants of the liver model. Cytokines were measured after treatment with control (Ctrl, 0.1% DMSO), 1 µM, 10 µM, 20 µM of TVX or LVX for 48 h and 72 h. (**a**–**e**) Bars indicate cytokine concentrations (pg/mL) plotted as ratio to control (RTC, dotted baseline) and represent mean ± SD of at least 4 independent biochip experiments with at least 3 different MDM donors (n ≥ 4). *p ≤ 0.05, **p ≤ 0.01, ***p ≤ 0.001 (Multiple t tests with Holm-Sidak’s multiple comparison test).

Previous studies in rodents revealed that TVX hepatotoxicity is linked to a detrimental immune-response^36^. Therefore, inflammatory responses after drug administration were measured by quantification of cytokines in sampled vascular supernatants (Fig. 6). Treatment with 10 µM TVX resulted in an increased release of tumor necrosis factor-alpha (TNF-α) (48 h, 72 h), interleukin (IL)-18 (48 h) and IL-8 (48 h) compared to control-treated models (Fig. 6a, d and e). Moreover, the release of IL-18 at 10 µM doses and IL-8 at 10 µM and 20 µM doses were significantly increased in contrast to LVX (Fig. 6d and e). 20 µM TVX further induced a rise in TNF-α (48 h, 72 h) and IL-1β (48 h) (Fig. 6a and b). In addition, administration of 20 µM TVX elicited a significant decrease in IL-6 in the vascular chamber after 48 h and 72 h, which was also significant in comparison to LVX (Fig. 6c). In contrast, stimulation of liver models with LVX did not result in altered cytokine profiles compared to control models. Nevertheless, a slight increase in TNF-α was observed in models treated with 20 µM LVX after 72 h (Fig. 6a). The 24 h time point and corresponding lipopolysaccharide (LPS) controls for the verification of MDM activation are shown in supplementary data (Fig. S5).

Subsequently, it was investigated whether modulation of the immune response by TVX treatment is indeed dependent on integrated MDMs. For this purpose, the cytokine release in models with MDMs was compared to models without MDMs after treatment with 20 µM TVX for 72 h (Fig. S6). With the exception of IL-6, no significant differences were observed between models with or without MDMs. Only upward trends in IL-1β and IL-8 concentrations were indicated in models with MDMs after 48 h (Fig. S6b,d). Nevertheless, a clear dependence of IL-6 increase by integrated MDMs compared to models without MDMs was shown at all measured time points (Fig. S6c).

### Examination of glutathione depletion and mitochondrial ROS formation as toxicity mechanisms in drug-treated liver models

**Figure 7:**
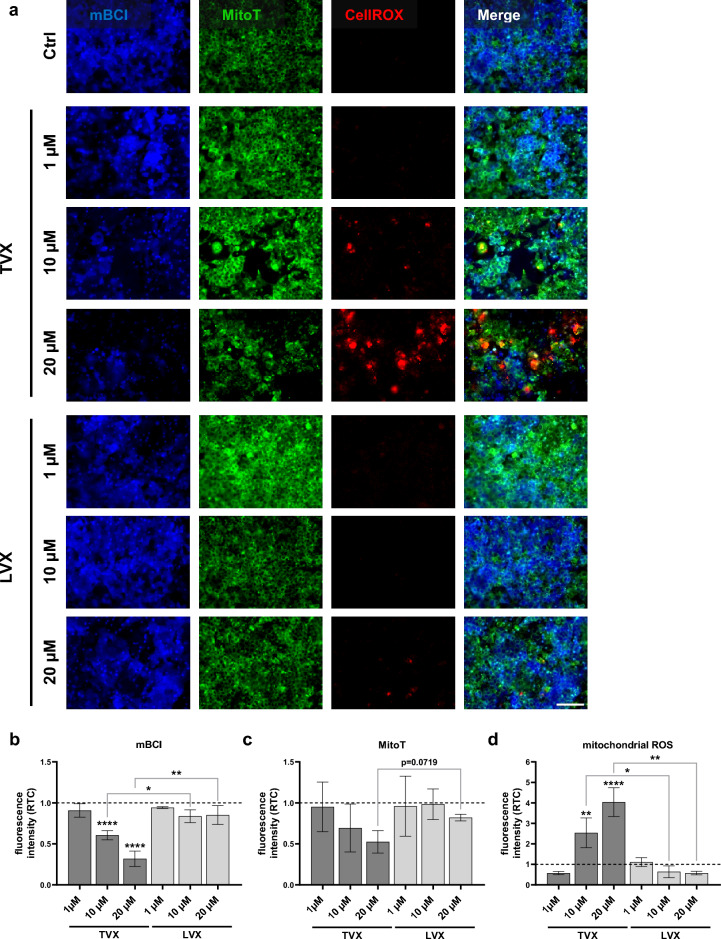
Live cell staining of hepatic cell layers in the liver model. (**a**) Representative images of glutathione level (mBCI, blue), mitochondrial integrity (MitoT, MitoTracker, green) and ROS formation (CellROX, red) after treatment with control (Ctrl, 0.1% DMSO), 1 µM, 10 µM and 20 µM TVX or LVX for 72 h in the liver model. Scale bar, 100 µm. (**b-d**) Fluorescence signal quantification of mBCI (**b**), MitoTracker (**c**) and mitochondrial ROS (**d**). Bars show fluorescence intensity plotted as ratio to control (RTC, dotted baseline) and represent mean ± SD of 3 independent experiments (n = 3 with 5 randomly selected membrane regions for each condition). *p ≤ 0.05, **p ≤ 0.01, ****p ≤ 0.0001 (One-way ANOVA with Dunnett’s multiple comparison test, two-tailed t test for comparison between indicated condition.

Recently, it has been shown in mice that mitochondrial ROS formation plays a major role in TVX-mediated hepatotoxicity^37^. We thus investigated this mechanism in our human liver model. Herein, treatment with either 10 µM or 20 µM TVX resulted in a reduction of intracellular glutathione in HepaRG cells, shown in representative images and fluorescence signal quantification (Fig. 7a and b). TVX induced a significant decrease in monochlorobimane (mBCI) signal intensity by 39% (10 µM) and 68% (20 µM) compared to control-treated models (Fig. 7b). TVX treatment significantly reduced the mBCI signal intensity compared to LVX at 10 µM and 20 µM doses. Furthermore, a concentration-dependent decline in the MitoTracker signal intensity was observed in TVX-treated cells, which was most remarkable at a concentration of 20 µM TVX compared to LVX (Fig. 7c). In addition, stimulation with 10 µM TVX induced a 2.5-fold increase in mitochondrial ROS and a fourfold elevation after treatment with 20 µM TVX compared to control treatment (Fig. 7d). Furthermore, ROS formation was significantly increased in models treated with 10 µM and 20 µM TVX compared to LVX at equal concentrations. A minor decline of mBCI and MitoTracker signal intensity was further demonstrated in models treated with higher concentrations of LVX (Fig. 7b and c), however these effects were marginal. Levels of mitochondrial ROS were not altered after LVX treatment compliant to control-treated models (Fig. 7d). Moreover, the reduction of glutathione and generation of mitochondrial ROS induced by TVX were not dependent on the presence of MDMs within the model. Both models with and without MDMs demonstrated an increase in ROS production and a concomitant decrease in glutathione (Fig. S7).

## Discussion

In the present study, the DILI potential of TVX and the structurally-related non-toxic compound LVX were investigated at human therapeutic concentrations in the liver model. Preclinical studies in mice failed to reveal the toxicity of TVX resulting in severe hepatic adverse events in human patients and withdrawal of the drug. By utilizing a human in vitro liver model, it was demonstrated that TVX, but not LVX, concentration-dependently impaired vascular and hepatic tissue morphology and cellular viability, while 2D monocultures of LSECs did not indicate TVX-induced toxicity. Interestingly, direct intrinsic hepatotoxicity was detected in 2D cultured HepaRG cells, which was, however, slightly increased in 3D liver models at the highest concentration of TVX. Due to the multicellularity and compartmentalization, the utilization of this 3D liver model can reveal off-target reactions such as toxicity to LSECs. Cellular damage induced by TVX in the 3D liver model was associated with an increased release of LDH and ALT. Interestingly, LDH and ALT elevations were exclusively detected in the vascular chamber. It is assumed that the induced cytotoxicity increases the cell layer leakage and facilitates a clearance of LDH and liver-specific ALT from the hepatic chamber towards the vascular circulation, which was validated by increased permeability from the hepatic chamber towards the vascular perfusion. The delayed elevation of ALT compared to LDH can be explained by the fact that it is exclusively released by hepatocytes in the model, whereas LDH is expressed ubiquitously and the vascular toxicity that occurs contributes to LDH accumulation. Nevertheless, it remains questionable whether vascular injury emerged prior to hepatocellular toxicity. Acetaminophen-induced injury of LSECs has already been described to precede hepatic toxicity, resulting in reduced sinusoidal perfusion and KC activation in mice^38^. At this point, it is tempting to speculate that the direct exposure of TVX to LSECs and MDMs through the vascular perfusion can in fact induce vascular injury prior to hepatocellular toxicity. To the best of our knowledge, it is not yet clear whether TVX induces direct cytotoxicity or whether metabolization and emerging toxic intermediates could primarily result in hepatocellular injury.

Moreover, the findings were consistent with previous results from Nguyen et al., who evaluated the toxicity of TVX and LVX in a bioprinted human liver model^24^. They observed liver injury at therapeutic concentrations ≥ 4 µM TVX, which was indicated by decreased ATP levels after 7 days of treatment. In line with our results, LVX did not induce ATP changes and models maintained cellular integrity up to 100 µM doses. By introducing native LSECs and tissue-resident MDMs in the model, we were able to advance this model presented by Nguyen et al. towards a more organotypic model. A transwell 3D liver co-culture model including non-parenchymal cells and human primary hepatocytes showed that treatment with TVX ≥ 25 µM resulted in increased LDH release after 8 days with associated decrease in cellular viability^39^. Compared to this study, TVX-induced toxicity was already observed in our model within 72 h at lower concentrations emphasizing the high sensitivity to TVX.

Even though TVX is widely known as a potent DILI-inducing compound, the underlying mechanisms remain mostly elusive. Studies in rodent models already indicated TVX-induced tissue injury upon co-stimulation with LPS^19,40,41^. Moreover, synergistic interaction of TVX and TNF-α resulted in concomitant increase in pro-inflammatory cytokines causing hepatotoxicity in mice^42,43^. A further study showed that TVX inhibited the chemotactic movement of phagocytosing neutrophils and monocytes and thereby increased circulating TNF-α concentrations^44^. One of the underlying mechanisms identified in a follow-up study was the inhibition of apoptotic signaling in hepatocytes upon TVX application^45^. Previously, inhibition of the pannexin 1 channel (PANX1) by TVX was linked to dysregulated cell fragmentation during apoptosis^46^ explaining the decreased infiltration of immune cells toward find-me signals from apoptotic hepatocytes^45^. Further investigation revealed that TVX with TNF-α or LPS perturbs NF-κB-mediated transcription and prolonged activation of MAPKs resulting in increased cytokine production and apoptosis^47^.

A major advantage of the liver model used in this study is that TVX toxicity in vascular and hepatic cell layers was clearly demonstrated independently from external LPS or TNF-α stimulation. Likewise, several in vitro studies have recently suggested a hepatotoxic effect of TVX without LPS using different liver models^24,25,27,39^ revealing direct KC activation by TVX and the involvement of intrinsic toxicity mechanisms. We were able to prove that TVX treatment triggered a concentration-dependent increase in pro-inflammatory cytokines IL-1β, TNF-α, IL-8 and IL-18 inducing a pro-inflammatory state in the model. Particularly, the pro-inflammatory cytokine TNF-α is widely known to be a proximal mediator of hepatocyte apoptosis and necrosis in inflammatory DILI^48,49^, which could have contributed to hepatic injury in the model. Apart from that, a concentration-dependent reduction of IL-6 was detected after TVX treatment. The suppressive effect of TVX on IL-6 secretion has already been demonstrated in 3D spheroid co-cultures of primary human hepatocytes and KCs^25^ and rat hepatocyte-KC models^50^, but only upon LPS co-stimulation. IL-6 is a pleiotropic cytokine that is crucial for hepatocyte homeostasis and proliferation^51^. In a previous study, it was shown that IL-6 plays a key role in hepatoprotection against acetaminophen-induced liver injury^52^. Furthermore, IL-6 appears to be a pivotal factor in reducing acute carbon tetrachloride (CCL4)-induced liver injury by amelioration of liver fibrosis and promotion of liver regeneration^53^. In relation to these findings, our study also indicates that the reduction of IL-6 is associated with a loss of the hepatoprotective function. In brief, it is suggested that an elevated secretion of pro-inflammatory cytokines and diminished hepatoprotection by IL-6 might contribute to TVX-induced hepatotoxicity in the model, whereas the non-DILI reference drug LVX did not alter the cytokine profiles.

Interestingly, the proposed immunomodulation of TVX was not exclusively mediated by integrated MDMs. The administration of 20 µM TVX in models with MDMs showed, except for IL-6, no difference in the cytokine release compared to models without MDMs (Fig. S6). A recent study was able to detect TVX-induced toxicity in a co-culture model of primary human hepatocytes and KCs after 5 days of treatment^25^. Interestingly, models including KCs showed a significantly higher toxicity after 48 h of treatment with TVX compared to models without KCs demonstrating a distinct role of KCs in the potentiation of TVX toxicity. However, this effect became marginal after 5 days. Thus, there is evidence that the role of integrated KCs in mediating TVX-induced toxicity is diminished during a longer treatment period and that intrinsic mechanisms are further involved in mediating toxicity. In comparison to this, we could not clearly indicate the dependence of the TVX-induced inflammation on the integrated MDMs in the liver model. This suggests that the induced immune response is modulated by other cell types, such as LSECs and hepatocytes and their intercellular cross-talk within the liver model. There are many indications that LSECs can contribute to the secretion of IL-1β, IL-6, TNF-α and IL-18 during cellular injury or in response to inflammatory stimuli^54–57^. Similarly, the capability of hepatocytes to secrete IL-8, IL-6 and TNF-α was demonstrated before^58–61^ leading to the hypothesis, that the TVX-induced immune response is orchestrated by a multicellular cytokine release and is not solely dependent on the presence of MDMs. We therefore conclude, that LSECs and hepatocytes may play a more crucial role in the immune-mediated mechanism of TVX than previously anticipated. To further elucidate the involvement of LSECs in the proposed immune response, additional experiments could be performed with and without LSECs. Due to the permeability of the integrated membrane with randomly distributed pores, this analysis was not possible. Hepatocytes are very sensitive to shear stress^62^ and further undergo a decrease in metabolic activity when cultured alone^28^. The absence of a LSEC lining exposes the hepatocytes to greater shear forces and elevated membrane deflection, which made this analysis unfeasible. Vice versa, single perfused LSEC culture without hepatocytes is not feasible for several days to investigate effects on LSECs alone. LSEC performance strongly benefits from growth factors such as VEGF secreted by hepatocytes^63^ and lack thereof presumably results in fast cell loss.

In addition, a concentration-dependent reduction of intracellular glutathione levels and an elevation of mitochondrial ROS formation within the hepatic tissue was demonstrated after treatment with TVX. These results are consistent with a previous microarray analysis using primary hepatocytes, which indicated a dysregulation of mitochondria-associated genes and impaired mitochondrial functionality in cells treated with TVX compared to other quinolone agents^64^. Besides, treatment of HepG2 cells with TVX resulted in glutathione depletion and concomitant increase in oxidative stress. No differences in glutathione levels were indicated in treated rats due to species-specific variation in gene expression. Moreover, an in vivo study using a heterozygous superoxide dismutase (Sod) 2^+/-^ mouse model and immortalized HC-04 hepatocytes, demonstrated an elevated mitochondrial production of peroxynitrite (ONNO^-^) after administration of the analogous prodrug alatrofloxacin^37^. By using the microphysiological liver model described here, it was possible to demonstrate previously suspected toxicity mechanisms and to uncover new pathways of TVX-mediated toxicity.

A major advantage of organ-on-chip consists in its scalable biological complexity for co-cultured cell types to precisely dissect molecular and cellular targets of cytotoxicity in a controllable manner. The cellular sources can be selected depending on its application. In the liver model, proliferating HepaRG cells were used as an adequate alternative to human primary hepatocytes, the "gold standard" for in vitro drug testing, due to their availability, comparable CYP enzyme expression and presence of relevant efflux and uptake transporters^65–67^. In comparison, other hepatic cell lines, such as HepG2, express significantly lower metabolic activity compared to HepaRG cells and are therefore not suitable for in vitro toxicity testing^65,68^. Furthermore, primary human hepatocytes are prone to rapid dedifferentiation and detachment after only a few days, which renders them unsuitable for longer culture periods of 7 days or longer^69^. On the other hand, HepaRG cells maintain their proliferation capacity and have further the ability to differentiate into hepatocyte-like cells and cholangiocytes-like cells and thus mimic the in vivo liver microanatomy more closely^32^. Besides choosing suitable hepatocytes, genetically modified upcyte LSECs were utilized as a surrogate to human primary LSECs. Recently, we demonstrated that upcyte LSECs maintain characteristics of primary LSECs, such as expression of specific surface markers, uptake of macromolecules and morphological integrity highlighting their suitability for in vitro hepatotoxicity testing^29^. By using upcyte LSECs, frequently observed problems such as rapid de-differentiation and low proliferation capacity in primary human LSECs^70^ were circumvented without compromising on functionality and morphology. Moreover, MDMs were incorporated in the liver model as an alternative cell source for human Kupffer cells. The convenience of obtaining MDMs by PBMC isolation allowed us to obtain a higher number of different donors with high availability and reproducibility. In comparison, primary Kupffer cells are less frequently available, show significant batch-to-batch variability and are often contaminated with other non-parenchymal cell fractions^71^.

Nonetheless, compared to primary cells, cell line-based models do not allow the investigation of patient risk factors and variability. Furthermore, future studies should focus on the establishment of isogenic models incorporating human induced pluripotent stem cells (iPSCs) to minimize allogenic effects^72^.

At this point, it should be mentioned that although there are at least three biological replicates for all experiments, the actual number of some experiments was much higher due to different experimental read-outs. Further, experiments were performed at separate time points from different vials or cell culture passages. Nevertheless, the hepatotoxic potential of TVX was clearly distinguish from its non-toxic analogue LVX. For less distinct drug-induced adverse effects, however, the number of replicates should be increased.

In summary, TVX-induced DILI was detected in the liver model at concentrations comparable to human therapeutic doses. The model was capable to reveal TVX toxicity without co-stimulatory LPS or TNF-α administration, demonstrating the superiority of the model compared to rodent models. The findings contribute to the understanding of immunomodulatory and intrinsic toxicity mechanisms of TVX and verified the non-DILI profile of LVX. Therefore, we emphasize the potential of the human liver microphysiological model as an in vitro platform for the evaluation of DILI. The model could be relevant to address limitations of conventional animal models and could prove useful to detect undesirable drug adverse events to avoid unnecessary animal suffering during preclinical testing.

## Materials and methods

### Biochip fabrication

Biochips were manufactured by Dynamic42 GmbH (Jena, Germany) from injection-molded polybutylene terephthalate base bodies. Biochips consist of an upper vascular and lower hepatic cultivation chamber. The upper and lower chamber, including channels, have an area of 2.18 cm^2^ and 1.62 cm^2^, respectively. The total volume, considering both inlet and outlet, is 290 µL for the upper chamber and 270 µL for the lower chamber. For cell seeding, a volume of 200 µL is used in the upper chamber and 150 µL in the lower chamber. Both chambers were separated by an integrated 12 µm thick polyethylene terephthalate (PET) membrane with a pore density of 1 × 10^5^ pores/cm^2^ and a pore diameter of 8 µm (TRAKETCH Sabeu, Radeberg, Germany). Microfluidic vascular reservoirs (Mobitec, Göttingen, Germany) were attached to the inlets of the vascular chamber and served as a reservoir for culture perfusion medium. The biochips were connected by PharMed BPT thermoplastic elastomer tubing (ISMATEC, Wertheim, Germany) and were perfused using peristaltic pumps (ISMATEC).

### Cell culture

All cultivation and incubation procedures were performed at 37 °C and 5% CO_2_ in a humidified cell culture incubator, unless stated otherwise. Cells used in this study were tested negative for mycoplasma.

Undifferentiated HepaRG cells were obtained from Biopredic International (Rennes, France) and were cultured as described before^73^. Cells were seeded at a density of 2.7 × 10^4^ cells/cm^2^ in culture flasks and were cultured in William’s Medium E (GIBCO, Darmstadt, Germany) containing 10% (v/v) fetal calf serum (FCS) (Capricorn Scientific, Ebsdorfergrund, Germany), 2 mM L-glutamine (Thermo Fisher Scientific, Darmstadt, Germany), 5 µg/mL insulin (Sigma-Aldrich, Steinheim, Germany), 50 µM hydrocortisone-hemisuccinate (Sigma-Aldrich) and 1X antibiotic antimycotic solution (AAS) (Sigma-Aldrich). The cells were cultured for two weeks with medium exchange every 3–4 days before HepaRG differentiation was initiated. Differentiation was induced by adding 2% dimethyl sulfoxide (DMSO) (Sigma-Aldrich) to the culture medium for at least two weeks as previously described^32,73^. Only fully differentiated HepaRG cells between passage 15–20 were used for biochip experiments.

Expandable human upcyte liver sinusoidal endothelial cells (LSECs) were purchased from upcyte technologies (Hamburg, Germany). The cells were freshly thawed and seeded at a density of 1.3 × 10^4^ cells/cm^2^ on collagen A (PAN-Biotech, Aidenbach, Germany)-coated culture flasks. LSECs were cultured in supplemented endothelial cell growth medium (ECGM) MV (Promocell, Heidelberg, Germany) with AAS. LSECs were freshly thawed for every experiment from a new cryovial and were not expanded further to prevent dedifferentiation.

Human peripheral blood mononuclear cells (PBMCs) were isolated from blood of healthy human donors by density gradient centrifugation. For each independent chip experiment a new blood donor was chosen. The study was approved by the ethics committee of the Jena University Hospital (2020-1684, 3939-12/13). All donors were informed about the aim of the study and gave written consent. All procedures were performed according to the approved guidelines and regulations and to the guidelines set forth in the Declaration of Helsinki. The blood was separately drawn into S-Monovette K3 EDTA tubes (Sarstedt, Nümbrecht, Germany) for isolation of PBMCs and S-Monovette Serum Gel tubes (Sarstedt) to obtain autologous serum. Autologous serum was directly extracted by centrifugation at 2000 g for 10 min at room temperature (RT) and was stored at − 20 °C until usage. For the isolation of PBMCs, blood from the same donor was pooled and diluted with an equal volume of PBMC isolation buffer containing 0.1% bovine serum albumin (Carl Roth, Karlsruhe, Germany) and 2 mM ethylenediaminetetraacetic acid (EDTA) (Sigma-Aldrich) in phosphate buffered saline (PBS) without calcium and magnesium (Lonza, Cologne, Germany). The blood isolation buffer mixture was gently overlayed on lymphocyte separation medium (Capricorn). The layered solution was centrifuged at 800 g for 20 min at RT without brakes. Precipitated lymphocytes were collected from the interphase and were subsequently washed with cold PBMC isolation buffer (4 °C). The cell suspension was centrifuged at 200 g for 8 min at 4 °C without brakes. The PBMCs were washed with cold PBMC isolation buffer again and were centrifuged at 150 g for 8 min at 4 °C with brakes. PBMCs were resuspended and seeded into 6-well plates at a density of 1 × 10^6^ cells/cm^2^ for adhesion-dependent monocyte enrichment. PBMCs were cultured in X-VIVO 15 medium (Lonza) with 10% (v/v) human autologous serum, AAS, 10 ng/mL macrophage colony-stimulating factor (M-CSF) and 10 ng/mL granulocyte-macrophage colony-stimulating factor (GM-CSF) (Peprotech, Hamburg, Germany) to induce macrophage differentiation. Cells were washed twice with X-VIVO 15 medium after 1 h of incubation to remove the non-adherent cell fraction. Remaining adherent monocytes were differentiated to MDMs in culture and on-chip for at least 5 days.

### Liver model assembly

Biochip chambers were gradually seeded with LSECs, MDMs and differentiated HepaRG cells (Fig. S8) on collagen A-coated membranes as previously described^28^. The following cell densities were selected based on cell number ratios found in the liver. The liver consists of approximately 60% hepatocytes, 19% LSECs and 15% Kupffer cells^74^. Based on this, HepaRG cells, LSECs and MDMs were seeded at comparable percentages of 60%/ 20%/ 20%, respectively. Concisely, human LSECs were seeded in the vascular chamber at a density of 0.45 × 10^5^ cells/cm^2^ (1 × 10^5^ cells in total) and were cultured in supplemented endothelial cell growth medium (ECGM) MV with AAS until reaching confluency. Medium was exchanged to vascular perfusion medium (VPM) comprising Medium 199 (Biozym, Hessisch Oldendorf, Germany), 5% (v/v) human autologous serum, 680 µM L-glutamine, 25 µg/mL heparin (Sigma-Aldrich), 5 µg/mL ascorbic acid (Thermo Fisher Scientific), 10 ng/mL M-CSF, 10 ng/mL GM-CSF and AAS prior to the seeding of MDMs. Human MDMs were seeded on top of the LSECs at a density of 0.45 × 10^5^ cells/cm^2^ (1 × 10^5^ cells in total) in VPM. MDMs and LSECs were cultured for 24 h under static conditions. Following this, differentiated HepaRG cells were seeded in the opposite hepatic chamber at a density of 1.85 × 10^5^ cells/cm^2^ (3 × 10^5^ cells in total) in hepatic thawing and seeding medium containing William’s Medium E, 5% (v/v) FCS, 4% hepatocyte thawing and plating supplements CM3000 (GIBCO), 1 µM dexamethasone (GIBCO), 2 mM L-glutamine, 5 µg/mL insulin, 5 µM hydrocortisone-hemisuccinate and AAS. Adhesion of the cells to the membrane was facilitated by overhead incubation of the biochip for 24 h. Subsequently, HepaRG cells were cultured as a hanging layer. The hepatic thawing and seeding medium was replaced by hepatic perfusion medium composed of William’s Medium E, 10% (v/v) FCS, 3.6% hepatocyte maintenance supplements CM4000 (GIBCO), 0.1 µM dexamethasone, 2 mM L-glutamine, 5 µg/mL insulin, 5 µM hydrocortisone-hemisuccinate, 0.1% DMSO and AAS after 24 h. The model was cultured for 24 h under static conditions with medium exchange in both chambers before applying vascular perfusion. Liver models were perfused via the vascular chamber at 25–50 µL/min (equals 0.3–0.7 dyn/cm^2^; 0.03–0.07 Pa). The perfusion rate was accordingly selected to microvascular shear stress of 0.1–0.5 dyn/cm^2^ found in the human liver sinusoid in vivo^28,75,76^. Models were pre-perfused for 24 h prior to initial drug treatment.

### 2D cultures

Pre-differentiated HepaRG cells or LSECs were seeded on collagen A-coated 96-well plates. The same cell densities were used analogous to 3D liver models. HepaRG cells were seeded at density of 1.85 × 10^5^ cells/cm^2^ (0.6 × 10^5^ cells in total) in hepatic thawing and seeding medium and LSECs were seeded at a cell density of 0.45 × 10^5^ cells/cm^2^ (0.15 × 10^5^ cells in total) in ECGM MV with AAS. After 24 h of culture, the medium was changed to hepatic perfusion medium for the HepaRG cells (analogous to perfusion experiments). HepaRG cells and LSECs were pre-cultured with daily medium exchange for 2 days to reach confluency before starting drug treatment.

### Drug stimulation

Drug concentrations were selected in relation to previously determined clinical human maximum plasma exposure (C_max_) values of trovafloxacin and levofloxacin^25,27,77^, which are consistent with values from pharmacokinetic studies of single oral drug doses^78–81^. Briefly, trovafloxacin mesylate and levofloxacin (Sigma-Aldrich) were solubilized in DMSO to 20 mM stock solutions. The stock solutions were diluted in VPM to the respective working concentrations without exceeding a DMSO concentration of 0.1% to rule out unspecific solvent toxicity. A vehicle control was provided for every experiment by diluting DMSO in VPM to a final concentration of 0.1%. Staurosporine solution from *Streptomyces sp.* (Sigma-Aldrich) was diluted in VPM to a concentration of 10 µM and was used as a positive control to induce cytotoxicity. For assessment of macrophage activation and cytokine release, lipopolysaccharide (LPS) (Sigma-Aldrich) was diluted in VPM to a concentration of 100 ng/mL. Diluted compounds were administered daily within the vascular perfusion in 3D liver models for 3 days to assess acute toxicity or for 7 days to evaluate prolonged hepatotoxicity (Fig. S8). 2D cultured cells were treated with the diluted drugs over a period of 7 days with redosing during the daily medium exchange.

### Immunofluorescence staining

Immunofluorescence staining was performed after 7 days of drug treatment to investigate tissue integrity and presence of cell-specific markers. Membranes were excised from biochips and cells were fixed with ROTIHistofix 4% (Carl Roth) for 10 min at RT. Permeabilization and blocking was performed by adding PBS (Lonza) containing 0.1% saponin (Carl Roth) and 3% normal donkey serum (Abcam, Cambridge, UK) for 30 min at RT. The membrane was subsequently divided with scissors to independently stain the vascular and hepatic cell layers with the primary antibody solution. Primary antibodies ASGPR1 (BD Biosciences, Heidelberg, Germany), CYP3A4 (Sigma-Aldrich), α-GST (BIOZOL, Eching, Germany), CD32b (BIOZOL) and CD206 (Abcam) were incubated at 4 °C overnight. Membranes were washed with PBS/0.1% saponin and incubated with secondary antibodies DAPI (Thermo Fisher Scientific), donkey-anti-mouse-AF647 (Thermo Fisher Scientific), donkey-anti-rabbit-Cy3 (Jackson ImmunoResearch, Cambridgeshire, UK) and donkey-anti-goat-AF488 (Thermo Fisher Scientific) for 1 h at RT. Stained membranes were washed twice with PBS/0.1% saponin, once with PBS and lastly with AQUA AD iniectabilia (B. Braun, Melsungen, Germany). Samples were embedded in fluorescent mounting medium (Agilent Technologies, Waldbronn, Germany).

### Live cell staining

Biochips were removed from the perfusion after 72 h. Membranes were excised and incubated in phenol red-free William’s Medium E medium (PAN-Biotech) containing 100 µM monochlorobimane (mBCI) (Sigma-Aldrich), 5 µM CellROX Deep Red Reagent (Thermo Fisher Scientific) and 200 nm MitoTracker Red CMXRos (Thermo Fisher Scientific) for 30 min at 37 °C and 5% CO_2_. Membranes were washed with PBS and fixed with ROTIHistofix 4% for 10 min at RT. Samples were embedded in PBS on microscopic glass slides and imaged directly.

### Image acquisition and analysis

Fluorescence images were acquired as Z-stacks with 10 slices and a 0.49 µm optimal interval setting using the AxioObserver Z1 fluorescence microscope equipped with ApoTome.2 (Carl Zeiss AG, Jena, Germany). All images were taken with a Plan Apochromat 20x/0.8 M27 objective (Carl Zeiss AG). The membrane pores served as a reference for the orientation of the Z-stacks. Separation of the cell layers was performed by identifying specific nuclei size and shape and expression of cell-specific markers. Five images of randomly selected regions on the membrane were taken for quantification of fluorescence signal intensities and nuclei counts. Quantification was performed by using the cell image analysis software CellProfiler (Broad Institute, Cambridge, MA, USA). Briefly, a threshold (minimum cross entropy method) was applied to every image to minimize non-specific fluorescence background signals. Cell nuclei counts were quantified after declumping using the “IdentifyPrimaryObjects” function of CellProfiler. Fluorescence intensities were measured as total fluorescence intensity (TFI) after masking and extracting the fluorescence regions. Mitochondrial ROS generation was measured after applying a “parent-child relationship” to MitoTracker and CellROX channels in CellProfiler to determine signal overlap. The separated signal quantification of CD206 on LSECs and MDMs was performed using ImageJ (Fiji, NIH, Bethesda, MD, USA). MDMs were identified by significantly brighter staining than LSECs due to their naturally high expression of CD206. MDMs were outlined with regions of interests (ROIs) individually and mean fluorescence intensities (MFI) were extracted. No thresholding was applied due to high variances in staining intensities of the underlying LSEC layer. Comparable ROIs were applied to the LSEC layer to extract fluorescence values.

### Cytokine analysis

Cytokine release in vascular effluents was quantified using LEGENDplex Human Inflammation Panel 1 (BioLegend, San Diego, CA, USA). The assay was performed according to the manufacturer's instructions. Samples were measured using a BD FACSCanto II flow cytometer (BD Biosciences). Data analysis was performed using the LEGENDplex Data Analysis Software v8.0 provided by the manufacturer. Cytokines not shown in result figures were below the detection limit of the assay.

### Cell viability assay

Cellular viability of treated liver models was measured after 7 days of drug perfusion using the CellTiter-Glo Luminescent Cell Viability Assay (Promega, Madison, WI, USA). Biochip membranes were excised and transferred to a 48-well microplate containing cell-specific phenol red-free William’s Medium E medium. CellTiter-Glo Reagent was added at a ratio of 1:1. The microplate was shaken for 2 min on an orbital plate shaker and was afterwards incubated for 10 min at RT. The solution was transferred to a white 96-well microplate. The luminescence signal was measured in a luminescence plate reader (Tecan Reader infinite F201, Tecan, Männedorf, Switzerland).

### Analysis of LDH and ALT

LDH levels in vascular and hepatic medium supernatants were measured by using the Cytotoxicity Detection Kit^PLUS^ (LDH) (Roche, Basel, Switzerland). Therefore, samples were collected from drug-treated liver models every 24 h for up to 72 h. Supernatants were directly diluted 1:2 in LDH storage buffer containing 200 mM Tris (Carl Roth)-HCL (VWR International, Darmstadt, Germany) at pH 7.5, 10% glycerol (Sigma-Aldrich), 1% bovine serum albumin (Sigma-Aldrich) and AQUA AD iniectabilia to conserve LDH activity during freezing. Samples were stored at − 20 °C until performing the assay. Samples were thawed and the assay was performed according to the manufacturer’s instruction. LDH activity was analyzed spectrophotometrically at 490 nm and concentrations were calculated from a standard curve obtained from dilutions of the LDH standard (Tecan Reader infinite F201, Tecan).

The release of ALT into hepatic and vascular culture supernatants was analyzed using the Human ALT ELISA Kit (Abcam). The assay was performed according to the manufacturer’s protocol in a 384-well format with sample and reagent volumes accordingly adopted. ALT levels were determined spectrophotometrically at 450 nm and concentrations were calculated from an ALT standard curve (Tecan Reader infinite F201, Tecan).

### Statistical analysis

Statistical analysis was performed in GraphPad Prism software version 8.4.2 (GraphPad Software, La Jolla, CA, USA). Statistical tests with multiple comparison were performed as indicated in the figure legends. Statistically significant results are indicated in the figures as follows: *p < 0.05, **p < 0.01, ***p < 0.001, ****p < 0.0001.

### Supplementary Information

Supplementary Information.

## Data Availability

All data generated and analyzed during this study are included within this published article or its supplementary information.

## Abbreviations

AAS: Antibiotic antimycotic solution
α-GST: Alpha-glutathione s-transferase
ALT: Alanine aminotransferase
ASGPR1: Asialoglycoprotein receptor 1
CD206: Mannose receptor
CD32b: Fc fragment of IgG receptor IIb
CYP3A4: Cytochrome P450 3A4
DILI: Drug-induced liver injury
DMSO: Dimethyl sulfoxide
IL: Interleukin
KCs: Kupffer cells
LDH: Lactate dehydrogenase
LPS: Lipopolysaccharide
LSECs: Liver sinusoidal endothelial cells
LVX: Levofloxacin
mBCI: Monochlorobimane
MDMs: Monocyte-derived macrophages
PBS: Phosphate buffered saline
ROS: Reactive oxygen species
RT: Room temperature
TNF-α: Tumor necrosis factor-alpha
TVX: Trovafloxacin
VPM: Vascular perfusion medium
