# Supplementary material for: Evaluation of drug-induced liver toxicity of trovafloxacin and levofloxacin in a human microphysiological liver model
Source: Sci Rep. 2023 Aug 16;13:13338. doi: 10.1038/s41598-023-40004-z (PMC10432496; doi:10.1038/s41598-023-40004-z)
Supplement: Supplementary file 1 — Supplementary Information. [file 41598_2023_40004_MOESM1_ESM.docx]

**Evaluation of drug-induced liver toxicity of trovafloxacin and levofloxacin in a human microphysiological liver model**

Authors: Tim Kaden^1,2^, Katja Graf^1^, Knut Rennert^1^, Ruoya Li^3^, Alexander S. Mosig^2^, Martin Raasch^1*^

^1^ Dynamic42 GmbH, Jena, Germany

^2^ Institute of Biochemistry II, Center for Sepsis Control and Care, Jena University Hospital, Jena, Germany

^3^ Biopredic International, St Gregoire, France

^*^Corresponding author: Martin Raasch, Dr. rer. nat., Dynamic42 GmbH, Jena, Germany, Email: martin.raasch@dynamic42.com

**Appendix A. Supplementary data**


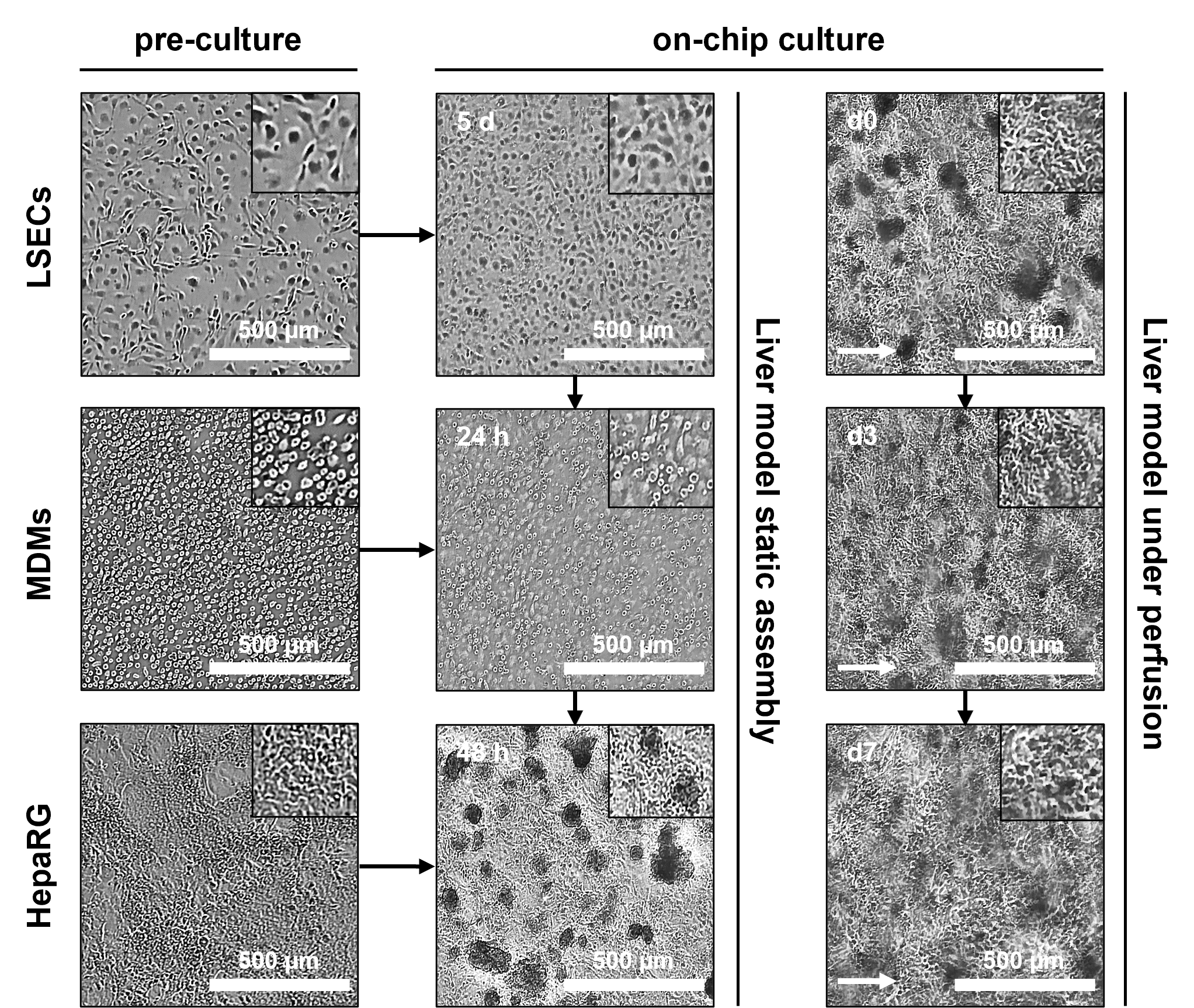


**Figure S1: Light microscopic monitoring of pre-culture, stepwise static model assembly and complete liver model under perfusion.** Representative images show pre-cultures of liver sinusoidal endothelial cells (LSECs, top, left row), human monocyte-derived macrophages (MDMs, middle, left row) and HepaRG cells (bottom, left row). All cell types were seeded and cultured statically in the Dynamic42 biochip (on-chip culture) for the indicated duration. LSEC layer in chip after 5 days (5 d) of static culture (top, mid row). MDMs on top of the LSEC layer 24 h after seeding (mid image). HepaRG cells after 48 h of seeding in the bottom chamber (bottom, mid row). Fully assembled models under flow were examined for intact cell integrity after 0 (top, right row), 3 (middle, right row) and 7 days (bottom, right row) (d). White arrows indicate the direction of flow. Scale bars, 500 µm.


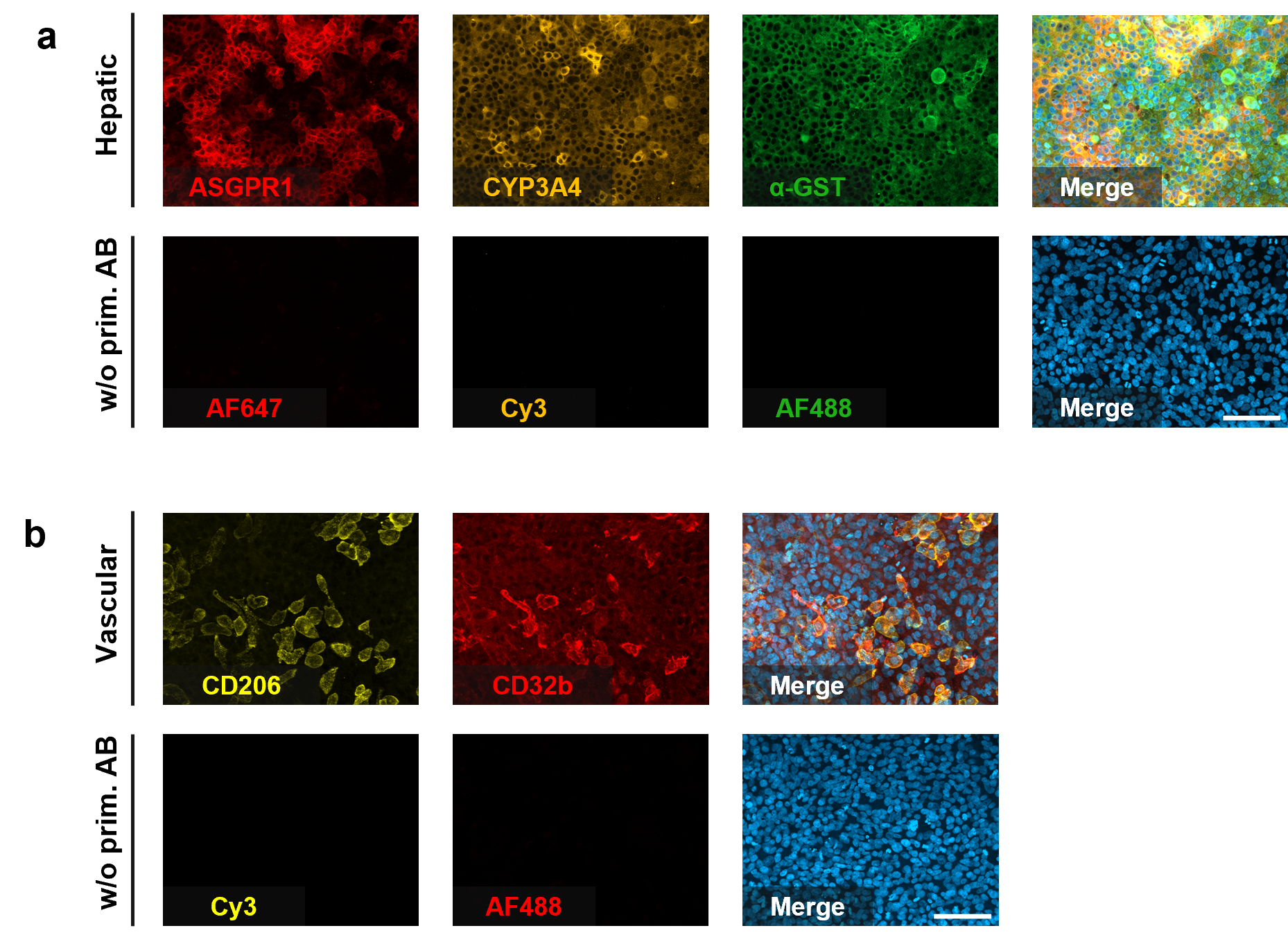


**Figure S2: Immunofluorescence staining for validation of primary antibody specificity.** Representative immunofluorescence images illustrate hepatic (a) and vascular (b) cell layers in liver models after 7 days of vascular perfusion with primary antibody (Hepatic/ Vascular) or without primary antibody (w/o prim. AB) staining. Hepatic (ASGPR1, CYP3A4, α-GST) and vascular (CD206, CD32b) markers are indicated for cell layers stained with primary and secondary antibodies. In cell layers stained only with the secondary antibodies, the respective fluorophores (AF647, Cy3, AF488) are shown in the figure. Merged images include all channels with nuclei (DAPI, blue). Scale bars, 100 µm.


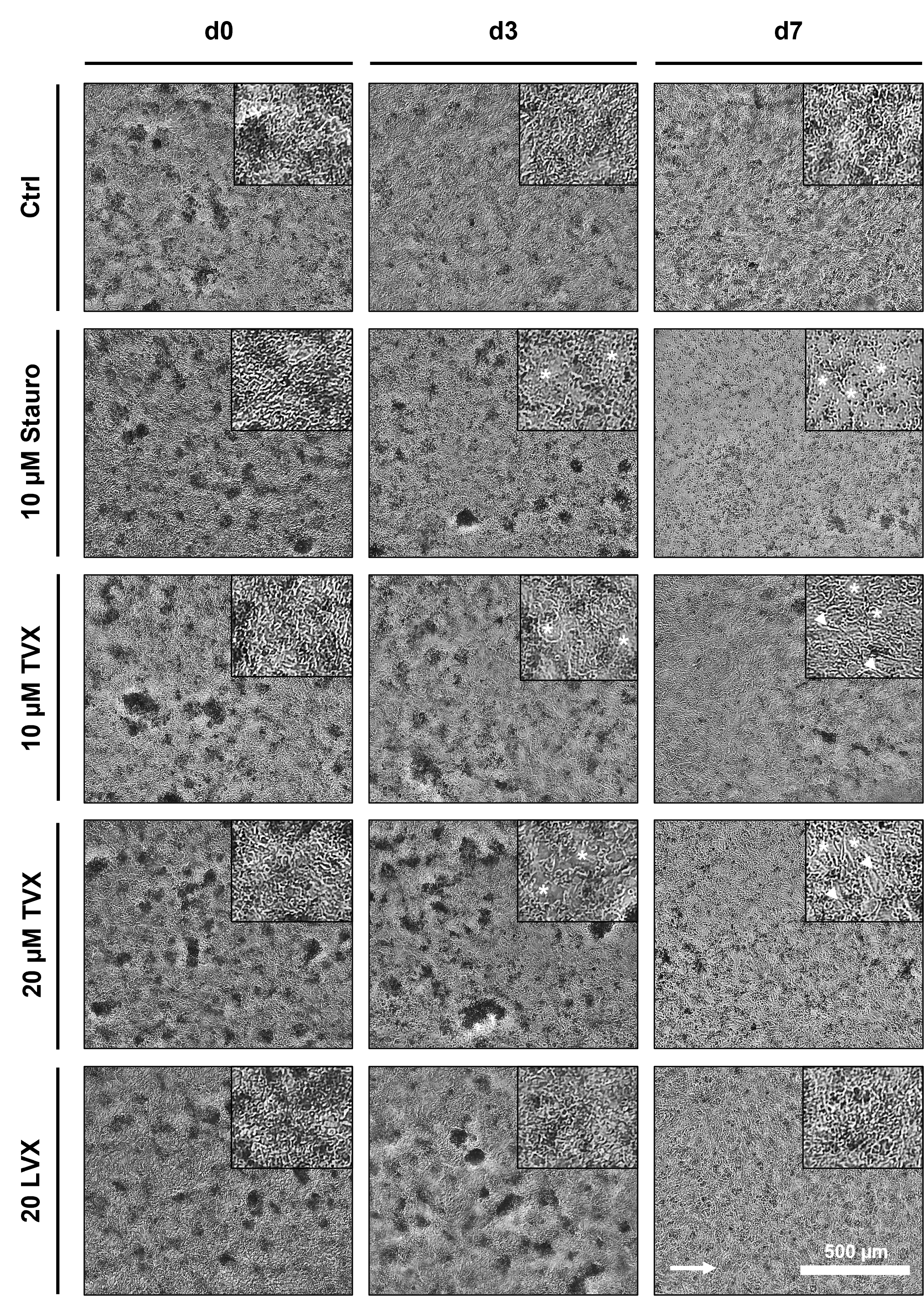


**Figure S3: Light microscopic monitoring of drug-treated liver models.** Representative images of liver models on day 0 (d0, before treatment), day 3 (d3) and day 7 (d7). Models were treated with control (Ctrl, 0.1% DMSO) or indicated drug concentrations of TVX and LVX for 7 days via the vascular perfusion. White asterisks show cellular detachment, and white arrow heads cell elongation. White arrow indicates the direction of flow. Scale bar, 500 µm.


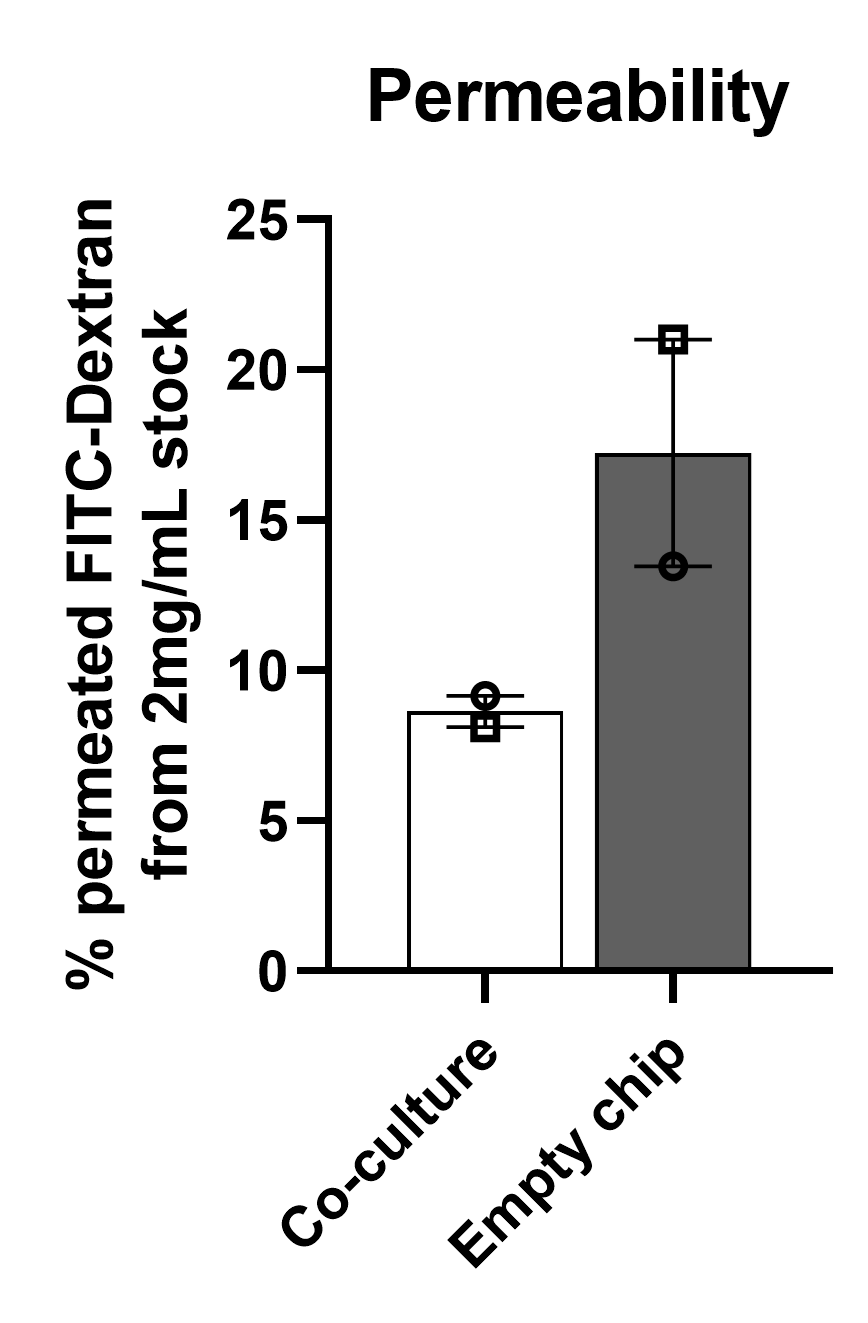


**Figure S4: Permeability between static hepatic chamber and perfused vascular chamber.** Fluorescein isothiocyanate-dextran (FITC-Dextran, 3-5 kDa, Sigma-Aldrich) was added at a concentration of 2 mg/mL into the static hepatic chamber of empty chips or a co-culture model containing LSECs (top chamber) and HepaRG (bottom chamber). Vascular perfusion with phenol red-free medium was applied for 1 h at 37 °C and 5% CO_2_ in a humidified cell culture incubator. Effluent samples from both hepatic and vascular chambers and vascular medium reservoirs were sampled and measured at 488/520 nm wavelength in a microplate reader. Values are plotted as mean percentages of vascularly permeated FITC-Dextran concentrations in relation to the 2 mg/mL stock solution. Error bars show mean ± SD of 2 independent chip cavities (n=2).

**a**

**b**

**c**

**d**

**e**

**j**

**i**

**h**

**g**

**f**

**Figure S5: Cytokine release in vascular supernatants of liver sinusoidal models.** Cytokines were quantified after treatment with control (Ctrl, 0.1% DMSO), 100 ng/mL LPS and 1 µM, 10 µM, 20 µM doses of TVX (a-e) or LVX (f-j) for 72 h. Bars indicate cytokine concentrations plotted as ratio to control (RTC, dotted baseline) and represent mean ± SD of at least 4 independent biochip experiments with at least 3 different MDM donors (n≥4). *p ≤ 0.05, **p ≤ 0.01, ***p ≤ 0.001, ****p ≤ 0.0001 (Multiple t tests with Holm-Sidak’s multiple comparison test).

**a**

**b**

**c**

**d**

**e**

**Figure S6: Cytokine profiles in presence (+MDMs) or absence (-MDMs) of MDMs in the liver sinusoidal model.** Vascular supernatants of liver models treated with 20 µM TVX for 72 h were analyzed. a-e) Interleaved bars represent cytokine concentrations plotted as mean of at least 3 independent biochip experiments with at least 2 different MDM donors (n≥3). *p ≤ 0.05 (Multiple t tests with Holm-Sidak’s multiple comparison test).


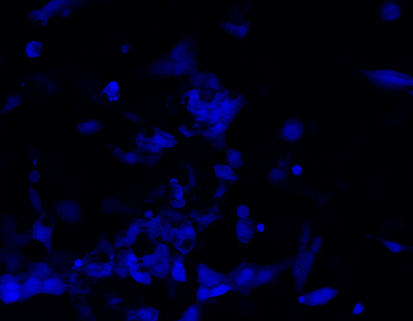


**mBCI**


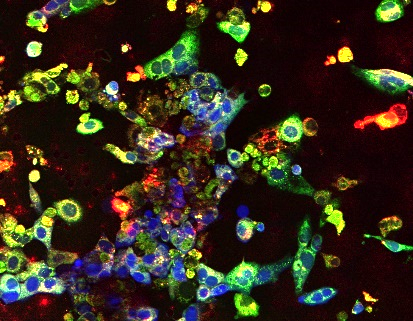


**Merge**


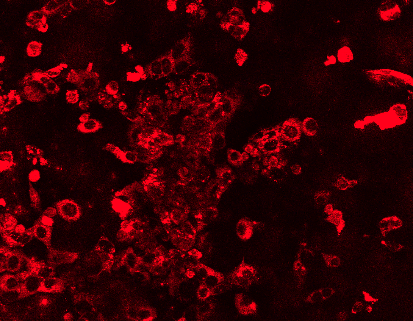


**CellROX**


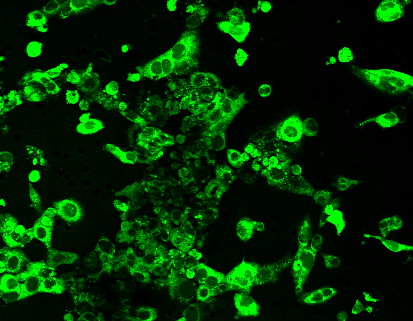


**MitoT**

**+ MDMs**

**TVX 20 µM**

**- MDMs**


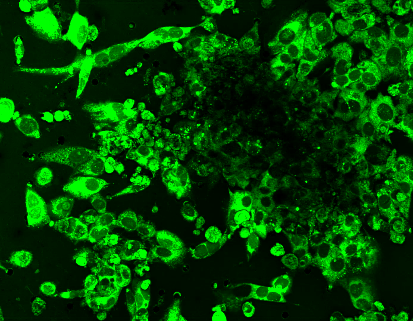

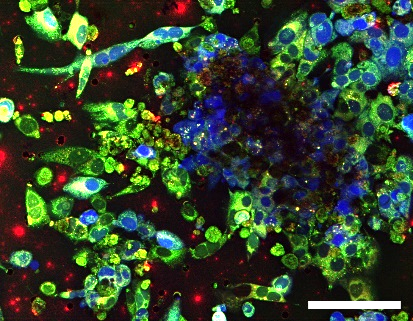

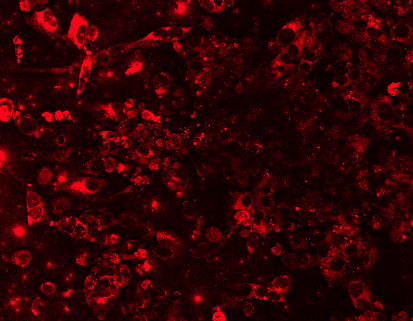

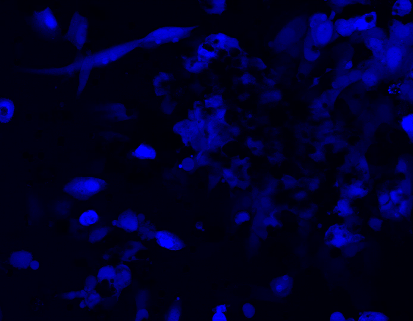


**Figure S7:** Live cell staining of hepatic cell layers in liver models with and without MDMs. Representative images of glutathione (mBCI, blue), mitochondrial integrity (MitoT, MitoTracker, green) and ROS formation (CellROX, red) in liver models with (+) or without (-) MDMs after treatment with 20 µM TVX for 72 h in the liver model. Scale bar, 100 µm.


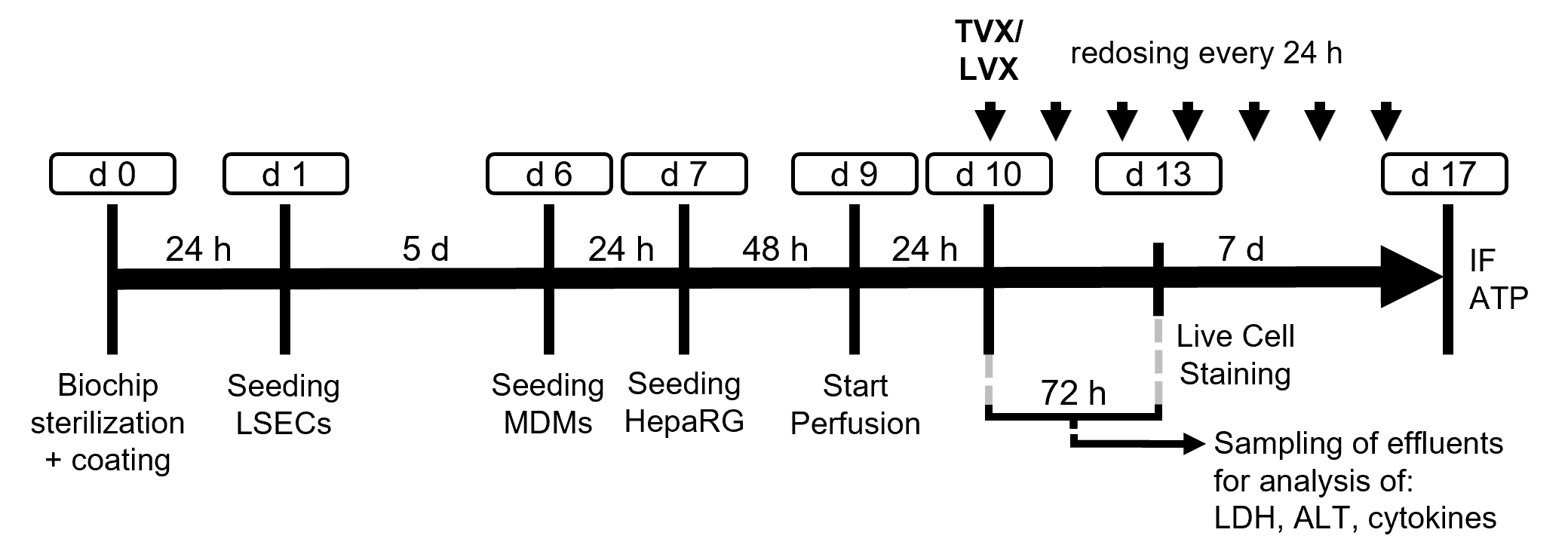


**Figure S8: Experimental timeline of liver model assembly, drug treatment, and analysis.** Biochips were sequentially seeded with liver sinusoidal endothelial cells (LSECs), monocyte-derived macrophages (MDMs) and hepatocytes (HepaRG). Models were statically cultured until day 9 and were pre-perfused for 24 h prior to treatment. Daily drug treatment with trovafloxacin (TVX) and levofloxacin (LVX) was started on day 10 for 72 h or 7 days. Endpoint assays included: sampling of effluents for measurement of lactate dehydrogenase (LDH), alanine aminotransferase (ALT), and cytokines with following implementation of live cell staining after 72 h or immunofluorescence (IF) staining and cell viability assay (ATP) after 7 days.
